# Landscapes of Urbanization and De-Urbanization: A Large-Scale Approach to Investigating the Indus Civilization’s Settlement Distributions in Northwest India

**DOI:** 10.1080/00934690.2018.1464332

**Published:** 2018-05-12

**Authors:** Adam S. Green, Cameron A. Petrie

**Affiliations:** University of Cambridge, Cambridge, UK

**Keywords:** Databases, GIS, urbanization, landscape archaeology, settlement archaeology, Indus Civilization

## Abstract

Survey data play a fundamental role in studies of social complexity. Integrating the results from multiple projects into large-scale analyses encourages the reconsideration of existing interpretations. This approach is essential to understanding changes in the Indus Civilization’s settlement distributions (ca*.* 2600–1600 b.c.), which shift from numerous small-scale settlements and a small number of larger urban centers to a de-nucleated pattern of settlement. This paper examines the interpretation that northwest India’s settlement density increased as Indus cities declined by developing an integrated site location database and using this pilot database to conduct large-scale geographical information systems (GIS) analyses. It finds that settlement density in northwestern India may have increased in particular areas after ca*.* 1900 b.c., and that the resulting landscape of de-urbanization may have emerged at the expense of other processes. Investigating the Indus Civilization’s landscapes has the potential to reveal broader dynamics of social complexity across extensive and varied environments.

## Introduction

Investigating transformations in the distribution and density of past settlements is crucial to the identification of “signature landscapes,” which are those generated by specific social, cultural, and economic processes within specific physical environments (Wilkinson 2003: 4–9). Comparative research has revealed an array of signature landscapes that have been associated with the emergence, transformation, and dissolution of social complexity across the globe (Algaze 2005; McIntosh 2005; Ur 2010; Wilkinson et al. 2014; Lawrence and Wilkinson 2015; Chase and Chase 2016; Lawrence et al. 2016, 2017). The identification and analysis of such landscapes contribute a large-scale dimension to models of social change, revealing interactions between societies and their dynamic and transforming environments. These investigations have the potential to transform these models, casting into high relief social processes that are dispersed across a broader landscape and may be hidden or obscured at the level of an archaeological excavation at a single site.

Patterns in settlement distribution, especially the frequency with which sites appear within a given area or environment, play a useful role in these studies by revealing settings that people favored as prevailing social conditions changed through time. However, archaeological surveys are also often constrained to specific areas by the logistics of fieldwork, limiting the scale of their interpretation and analyses. To investigate large-scale changes in settlement distribution, it is necessary to assemble and analyze large synthetic datasets built over many years by multiple teams (Lawrence and Bradbury 2012). Successfully integrating datasets requires recognizing the limitations and errors incumbent to the production of each constituent survey project.

**Figure 1.**
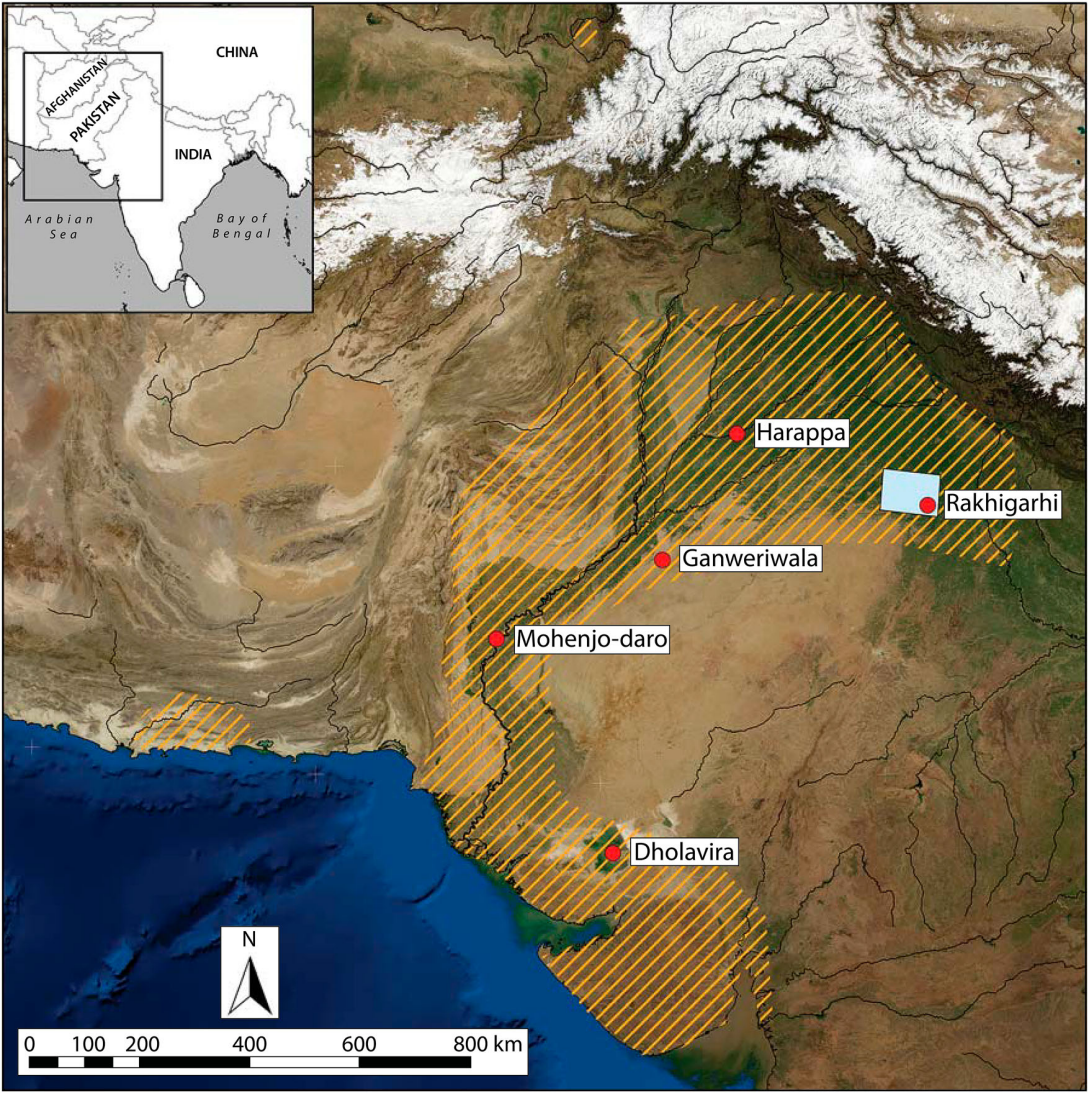
Geographical context and extent of the Indus civilization (orange lines). Sites that have been identified as cities (red dots) are shown as well as the sample area considered in this paper (blue square). Extent was derived from secondary sources. Basemap Source: http://earthobservatory.nasa.gov/Features/BlueMarble

Northwestern India was a key setting for the emergence of South Asia’s earliest complex society, the Indus Civilization. Indus cities arose around 2600 b.c. across extensive and ecologically diverse areas of western South Asia (figure 1), and concentrations of archaeological sites have been reported in the modern states of Rajasthan, Haryana, and Punjab in India (Stein 1942; Suraj Bhan 1975; Joshi et al. 1984; Possehl 1999; Shinde et al. 2008; Singh et al. 2008, 2010, 2011; Chakrabarti and Saini 2009; Dangi 2009, 2011; Kumar 2009; Pawar 2012). It has frequently been noted that the density of settlements across the alluvial plains of northwestern India appears to increase after ca. 1900 b.c. (Madella and Fuller 2006; Kumar 2009; Wright 2010: 317–318, 2012; Petrie et al. 2017). Climate change appears to have played a role in this shift, as changes in settlement density seem to have favored the variability of local environmental conditions in northwestern India in the face of a weakening in the Indian Summer Monsoon around 2200–2100 b.c. (Madella and Fuller 2006; Giosan et al. 2012).

The increase in settlement density in northwestern India may have been due to the strong possibility that this region received more reliable rainfall from a weakened monsoon (Petrie et al. 2017). As people left Indus cities, they appear to have populated particular areas, establishing new small-scale settlements and re-occupying mounds that had been abandoned in earlier periods. This apparent shift both resulted from and contributed to a process of de-urbanization, wherein smaller and more dispersed settlements replaced larger population aggregations. Much attention has been given to the process of urbanization that brought together multiple groups of specialized artisans and agro-pastoralists (Kenoyer 1997; Possehl 2002; Wright 2010). However, it is unclear how de-urbanization transformed these social relations, as it was a dispersed process that unfolded at a large number of sites across an extensive area, thus necessitating a large-scale approach that incorporates the results of multiple projects.

To utilize multiple datasets in aggregate studies, it is necessary to compare the approaches, questions, and methods that contributed to each researcher’s agenda (following Cooper and Green [2015]). It has been noted that site location reports from northwestern India vary in their intensity of survey coverage, adherence to modern administrative boundaries, and assumptions about the locations of past watercourses (Singh et al. 2008, 2010, 2011). To address these challenges, this paper describes the assembly of a pilot database that integrates all site location data from a sample region that encompasses two major surveys carried out by the “Land, Water and Settlement” project (Singh et al. 2010, 2011; Petrie et al. 2017). The data were then analyzed using geographic information systems (GIS) analyses; this was the first stage of a larger effort to integrate site locations from northwestern India into a single relational database, which is being carried out for the “TwoRains” project. This approach is informed by Kintigh (2006: 573), who has advocated increasing the scale of archaeological investigations without compromising the detail recorded in specific reports. It allows the analysis of site location data at different levels of certainty (following Lawrence and Bradbury [2012]). The pilot database facilitated a test of the following hypotheses: first, that in northwestern India, the Mature Harappan period saw the nucleation of settled population; second, that the Late Harappan period saw an increase in settlement density. Our results support these hypotheses and enhance the interpretation that site density increased in particular locations with the decline of Indus cities. It follows that the landscapes of urbanization and de-urbanization created by Indus populations integrated a range of varied environments to produce and sustain social complexity.

## Landscape Archaeology and the Indus Civilization

Landscape archaeology provides the approaches necessary to frame research on past social processes. It has been foundational to modeling social complexity in ancient Mesopotamia (Adams 1966, 1981; Adams and Nissen 1972; Wilkinson 2003; Ur 2010; Wilkinson et al. 2014; Lawrence and Bradbury 2012; Lawrence et al. 2016, 2017), and has also been critical to the study of complex societies across the globe (Kantner 2008; Chase et al. 2011; Glover 2012; Kosiba and Bauer 2012; Luo et al. 2014). Large scale analyses are necessary for outlining the interaction between emerging complex societies and their varied local settings, revealing patterns that are difficult to explain in reference to their local settings alone and thus must result from processes of greater regional integration (Lawrence et al. 2017). By incorporating data from locations across broad and varied environments, landscape approaches have the potential to challenge traditional models of complexity and urbanism. Such approaches have revealed processes such as the heterarchical clustering of settlements (for example, McIntosh [2005]) and alternative political trajectories (for example, Fargher and colleagues [2011]).

Wilkinson (2003: 4–9) argued that relationships between archaeological remains and their environmental contexts result in “signature landscapes” that exemplify the prevailing configurations of social, cultural, and economic processes within specific environmental settings and chronological periods. Signature landscapes can be compared to one another to investigate social change (Wilkinson 2003: 215). Site locations are key to this approach, but to address large-scale processes that take place throughout a landscape typically requires aggregating data built up by many projects. A framework for integrating heterogeneous survey datasets has been set out by Lawrence and Bradbury (2012), who characterize site locations using factors such as boundary certainty, geographical precision, and archaeological significance, ascertaining different levels of certainty in archaeological datasets. Boundary certainty addresses the size of archaeological sites and lies beyond the scope of this paper, but site location reports from northwestern India can be used to establish a basic level of certainty based on geographical precision (locations) and archaeological significance (approximate chronology). Linking multiple datasets has become essential to investigating shifts in settlement density that illustrate how populations engage with and retreat from local ecologies as social relations transform (Lawrence et al. 2017). This approach is particularly applicable to northwestern India, where integrating a wide range of site location reports has the potential to cast the Indus Civilization’s signature landscapes, and interrelationships between varied local environments and social complexity, into high relief.

### The Indus Civilization in northwestern India

After a protracted period of village-based occupation, the first cities in South Asia appeared during the Mature Harappan period of the Indus Civilization (ca. 2600–1900 b.c.), which were the largest of thousands of settlements across areas that today lie in western India and Pakistan (Marshall 1931; Wheeler 1953, 1966, 1968; Sankalia 1962; Fairservis 1967, 1971; Lal 1993, 1997; Kenoyer 1998; Chakrabarti 1999; Possehl 1999, 2002; Agrawal 2007; Wright 2010; Coningham and Young 2015; Ratnagar 2016). Five Indus sites are typically considered cities, and their locations in contrasting environments support the interpretation that they were to some degree politically discrete (Kenoyer 1997, 2006; Wright 2010; Petrie 2013; Sinopoli 2015; Petrie et al. 2017) (figure 1). At the same time, the aspects of Indus material culture that were shared across such a vast and varied extent suggest that the Indus Civilization’s political organization resulted in signature landscapes that were distinct from those materialized by other early complex societies. Excavations at Indus sites have produced evidence of a broad range of sophisticated technologies (K. K. Bhan et al. 1994; Vidale 2000; Miller 2007; Agrawal 2009), including copper metallurgy (Hoffman and Miller 2009), standardized weights and measures (Ratnagar 2003; Kenoyer 2010; Miller 2013), and engraved stamp seals (Joshi and Parpola 1987; Shah and Parpola 1991; Parpola et al. 2010; Green 2016). Indus settlements also present examples of civic coordination and planning, though they lack direct evidence for the extreme forms of social differentiation and political hierarchy reported in other complex societies (Wright 2010, 2016; Green 2018).

Landscape approaches and archaeological surveys have been essential to challenging past narratives that suggest that the Indus Civilization was socio-culturally uniform and homogeneous (Piggott 1950; Wheeler 1966). Initial surveys highlighted its great extent (Stein 1942; Sankalia 1962), and subsequent studies identified local variation in material culture (Suraj Bhan 1969, 1975; Mughal 1971; Possehl 1980; Possehl and Raval 1989; Possehl and Herman 1990). The increase in fieldwork in India between 1960 and 1980, predominantly recorded in *Indian Archaeology: A Review*, has been used by multiple researchers to generate site location lists. One such study by Joshi and colleagues (1984: 513) suggested that the distribution of site locations revealed “economic pockets” during the Mature Harappan period, which were apparent concentrations of settlements that were closely knit and perhaps economically self-sufficient. As features of the Urban Phase, economic pockets were thought to support one or more large settlements (Joshi et al*.* 1984: 514).

Smaller settlements, which have many of the same characteristics as the cities themselves, comprise the majority of Indus sites (Chakrabarti 1999; Wright 2010; Petrie 2013; Sinopoli 2015). Surveys of the settlement distribution along the Beas River in Pakistan’s Punjab revealed that the economic diversification and intensification apparent in assemblages from the city of Harappa is also apparent in the material assemblages of nearby smaller settlements (Wright et al. 2001, 2003). Other studies have used survey data to clarify site distribution patterns in other Indus regions, including Sindh in Pakistan (Flam 1993, 2013; Jansen 2002; Shaikh et al. 2003; Mallah 2008), and Gujarat in India (Possehl and Raval 1989; Possehl and Herman 1990; Shinde 1992; Sonawane and Ajitprasad 1994; Possehl 1999).

The plains of northwestern India are characterized by a range of alluvial environments, an absence of mineral resources, extensive irrigation farming, and numerous archaeological sites from all periods. Some site locations were initially reported as early as 1832, and relatively informal excavations at Indus sites in this region began in the early twentieth century (Possehl 1999; Lahiri 2006). Field methods and recording improved with the reinvigoration of the Archaeological Survey of India under Sir John Marshall, but remained rudimentary by modern standards (Lahiri 2006). Parts of what is now northwestern India were later explored by Stein (1942) and Ghosh (1952), who assumed that settlement densities in the region resulted from proximity to now-dry watercourses. Further surveys through the 1970s and 1980s brought to light many important Indus sites, including Mitathal and Rakhigarhi (Suraj Bhan 1975; Suraj Bhan and Shaffer 1978; Francfort 1985), and there were several attempts to collate these data (Joshi et al. 1984; Possehl 1999).

Unfortunately, the majority of these studies predate the use of global positioning systems (GPS), so there is a degree of imprecision in the reported site location coordinates (Petrie and Singh 2008; Singh et al. 2008). During the same period, excavations were also undertaken at the sites of Kalibangan (Thapar 1975; Lal 1979, 2003), Banawali (Bisht 1978, 1987, 2005; Bisht and Asthana 1979), and Mitathal (Suraj Bhan 1975). These excavations were essential to developing ceramic typologies for northwestern India, which typically include pottery vessel types and styles like those found at the cities of Harappa and Mohenjo-daro along with other types and styles with local characteristics. Subsequently, excavations were carried out at Rakhigarhi, which appears to have been urban in scale and complexity (Nath 1998, 1999, 2001; Shinde 2016), and the smaller sites of Bhirrana (Rao et al. 2004) and Kunal (Khatri and Acharya 1995). More recent excavations at Farmana have unearthed large mud-brick houses, a coordinated street plan, and an extensive cemetery, highlighting additional associations between elements of material culture found at other major Indus cities and local artifact styles (Shinde et al. 2011). Material culture assemblages from these sites are believed to correspond to the periods nested within the overarching chronology of the Indus Civilization, such as those employed by Meadow and Kenoyer (1997, 2003), Possehl (2002), and Wright (2010, 2012). These periods include the Early Harappan, Mature Harappan, and Late Harappan periods. Following the Indus Civilization comes a sequence of phases marked by distinctive pottery types, such as Painted Gray Ware. This framework is widely utilized in South Asian archaeology, though the attribution of many types and styles to specific periods is not straightforward (Parikh and Petrie 2017, in press).

Since 2000 there have been many surveys conducted in several states in northwestern India, including Haryana (Shinde et al. 2008; Dangi 2009, 2011; Parmar et al. 2013), Rajasthan (Pawar 2012; Pawar et al. 2013), and Punjab (Sharan 2018). Most archaeological surveys in northwestern India have employed a “village-to-village” methodology, wherein a survey team visits the contemporary villages within an administrative unit and asks local informants where archaeological materials can be found (see discussion of these methods in Singh and colleagues [2010, 2011]). The number of villages and intensity of agricultural land use therefore impact the results of these surveys. Many site locations are only readily accessible through secondary studies, which combine the primary results of published and unpublished survey projects, and which reinforce the notion that the region was home to several dynamic settlement concentrations, though they differ on specific interpretations. For example, Kumar (2009: 17) argued that settlement density in northwestern India increased markedly during the Late Harappan period, while Chakrabarti and Saini (2009: 77) suggested that the change in population between the Mature and Late Harappan periods was less dramatic, indicating that that migration from the declining cities may be unlikely.

It has been clear for some time that a high-resolution evaluation of these site location data will improve scholarly understanding of the processes of urbanization and de-urbanization that created and transformed the Indus Civilization’s signature landscapes. The “Land, Water and Settlement” (hereafter LWS) project produced two complementary site location datasets that can anchor data assembly projects: the Rakhigarhi Hinterland Survey and the Ghaggar Hinterland Survey. LWS focused on rural life in northwestern India, and expanded and refined a subset of site location datasets from this region (Singh et al. 2008, 2010, 2011; Petrie et al. 2017). The LWS surveys demonstrated that during the Mature Harappan period there was an overall reduction in settlement density that sustained the emergence of larger urban settlements like Rakhigarhi (Singh et al. 2010, 2011). During the Late Harappan period, the number of sites in northwestern India appears to increase, but these settlements are typically small in size (Madella and Fuller 2006; Kumar 2009; Singh et al. 2010). This transformation is likely associated with climate change, and it has been suggested that a weakening summer monsoon prompted communities in northwestern India to diversify their agricultural practices (Madella and Fuller 2006). However, it is clear that this diversity emerged well before cities and may have provided the risk buffering and mitigation necessary to maintain food surpluses in the face of climate change (Petrie et al. 2016, 2017; Petrie 2017; Petrie and Bates 2017).

New landscape approaches to the Indus Civilization have the potential to reveal how social complexity integrates vast and varied environments in the face of dramatic changes in social scale. However, the environmental and socio-cultural diversity and variation across the extensive region occupied by Indus populations inhibit the understanding Indus landscapes if site location reports remain confined to the spatial silos of individual studies. Assembling Indus site location reports into larger integrated databases creates an opportunity to critically assess settlement densities and identify research strategies that will increase certainty by revealing areas where data need to be reviewed and re-examined and locations that will benefit from additional survey.

More research on the diverse range of social processes that unfolded in early complex societies is needed. It is particularly critical to determine when transformations in past landscapes reinforce current models of social complexity, and when they demand the revision of traditional models, and the Indus Civilization is particularly important in the regard. Investigating the Indus Civilization’s signature landscapes may reveal how particular environments, and variation within them at smaller scale, interact with heterarchical social processes, such as those outlined by Crumley (1995) and McIntosh (2005). Moreover, most classic studies of site location data tend to emphasize the relationship between an early complex society and a particular environment, such as Wilkinson (2003). The Indus offers a fundamentally different challenge: an example of an extensive early complex society that encompassed a great range of different environments.

## Methods

Assembling archaeological survey data from northwestern India into a single relational database facilitates the comparison, quantification, and spatial analysis of heterogeneous datasets. Though there have been several attempts to synthesize northwestern India’s settlement distributions (Joshi et al. 1984; Possehl 1999; Chakrabarti and Saini 2009; Kumar 2009), the inherent limitations and discrepancies between datasets are rarely considered. Singh and colleagues (2008, 2010, 2011) noted that some reports omit precise coordinates, utilize inconsistent naming protocols, and only implicitly define their survey boundaries. Moreover, many of the primary surveys that underpin these datasets used modern administrative boundaries to delimit study areas (such as districts or blocks), and survey coverage is often strongly influenced by assumptions about the location of watercourse locations (Petrie et al. 2017). Combining “other people’s data” into larger datasets requires identifying comparable attributes across datasets and assembling them into formats that can be cross-referenced (Atici et al. 2012). Integrating site location data within a single relational database is the first step toward developing a cyber-structure that preserves the character of particular datasets (Cooper and Green 2015). Toward this end, this paper aggregates site location reports to generate a novel tabulation that integrates all previously reported site locations within a sample area.

### Sources

The site locations from four secondary studies (Joshi et al. 1984; Possehl 1999; Chakrabarti and Saini 2009; Kumar 2009) were digitized to provide initial tables for the pilot database. These studies analyzed overlapping geographical regions using multiple primary site location reports. The two earlier studies examine settlement patterns across the entire extent of the Indus Civilization (Joshi et al. 1984; Possehl 1999), and the two later studies selected areas that were assumed to be in proximity to past watercourses in northwestern India (Chakrabarti and Saini 2009; Kumar 2009). Some primary site locations have been reported by multiple sources.

A series of unpublished tables based on previous efforts to combine Indus site locations into an integrated database was also included in the pilot database. These started with Possehl’s (1999) tabulations, and incorporated an additional table of site locations developed as a Google Earth .kmz file by Randall Law. This .kmz file presented Possehl’s tabulation in a format that could be read by Google Earth and projected onto satellite imagery. Law enhanced this dataset by visiting many locations, adding to or adjusting their coordinates. Although it was not formally published, Law’s .kmz file was made available to the scholarly community, and contains important supplementary notes for many locations mentioned in the secondary studies. This table has undergone some cleaning and revision via a comparison between the Possehl and Law datasets (Cameron Petrie and Edward Cork, personal communication 2008).

Additional tables derived from recent primary site location reports were drawn from location reports from the LWS surveys (Singh et al. 2010, 2011), a survey of the Mansa district of India’s Punjab (Sharan et al. 2013) and a report of site locations in the districts of Fatehabad in India’s Haryana and Mansa and Sangrur in India’s Punjab (Dangi 2011). The LWS surveys employed GPS and aimed for complete coverage within their bounded study regions. The Rakhigarhi Hinterland Survey (RHS) investigated a circular area roughly within a 15 km radius surrounding the major Indus city of Rakhigarhi (Singh et al. 2010), while the Ghaggar Hinterland Survey (GHS) targeted a previously un-surveyed area around the middle course of an important watercourse that is largely known from remote sensing imagery (Singh et al. 2011). These LWS surveys prioritized questions about site and water catchments over administrative districts.

### Pilot database development

To assemble the pilot database, tables derived from the above sources were imported into a relational database using FileMaker Pro (v15), which facilitated the speedy examination of attributes from non-corresponding tables prior to developing related fields through comparison. After importing the selected tables, each site location was given a unique identifying value: the Pilot TwoRains Identification Number (ptr_id). The resulting ptr_id list was initially extensive, including over 10,000 entries. Moreover, overlap between the original tables resulted in significant duplication of entries. To reduce the ptr_id list, entries that shared a common location were reclassified, which reduced the number of ptr_ids. As records based on the same site location were linked to the same key ptr_id, it became possible to query information about the same location derived from multiple sources. Duplicates were then assigned the same ptr_ids by projecting the site table in a GIS (ArcGIS v10.4.1) and examining each location against ESRI’s World Imagery.

**Figure 2.**
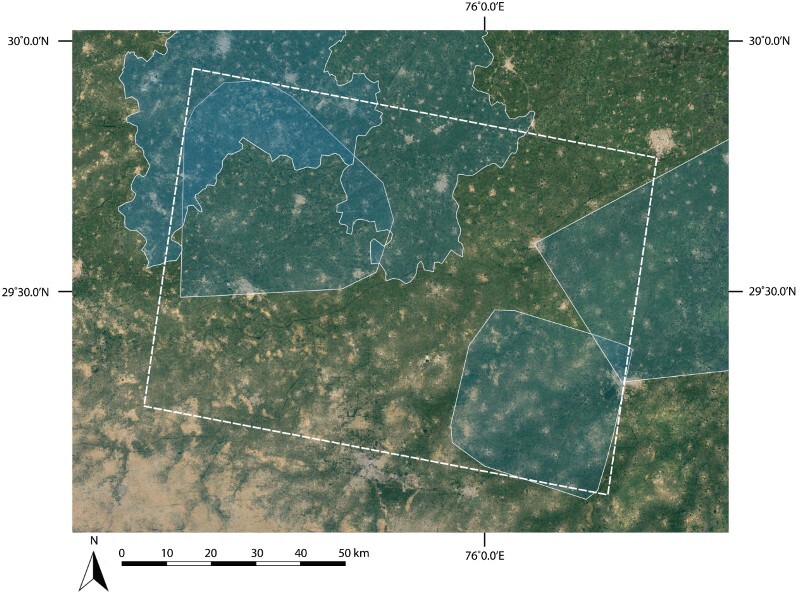
Primary studies that overlap the sample area (indicated by the dashed line). Secondary studies encompass the entire sample area (Joshi et al. 1984; Possehl 1999; Kumary 2009; Chakrabarti and Saini 2009). Basemap Source: Google Earth 2018.

While the resulting ptr_id table allowed the querying of related fields across multiple tables, standardizing the information available for each site location and reconstructing its history and characteristics required the review of each record. To evaluate settlement density in northwest India, ptr_ids from a sample area were selected for more detailed assessment. The sample area consists of a projected rectangle that encloses both LWS survey areas that was automatically generated (figure 2). In addition to the LWS site locations, the entire sample region was included within the research areas of all the major secondary studies of Indus Civilization site distribution mentioned above. The sample area encloses a projected area of 10476.77 km^2^ and includes 695 reported site locations.

Bibliographic information was assembled for each site location and cross-referenced with the original publications to the extent that primary sources were available, and assessments of site location accuracy and precision were included in the resulting table. Outright errors, reported locations that lacked complete geographical information, were located outside of South Asia, or were unlikely to be related to a specific location in the landscape, were flagged with the assistance of GIS analyses undertaken using ArcGIS 10.4.1 and QGIS v2.18.2. The apparent precision of site location reports was noted (also indicated by whether full geographical coordinates were included). Reported periodization for each site location was also compiled and included in the resulting table. The pilot database compiled the history of study for each site location, along with its earliest likely discovery date, and the tabulated results of this compilation are presented in the supplement accompanying this paper (supplemental material 1).

## Results

**Figure 3.**
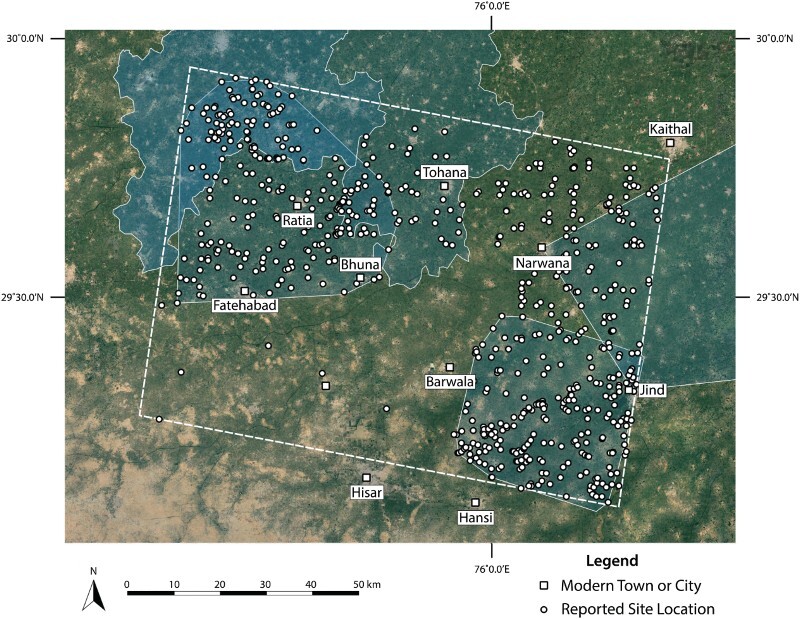
Distribution of site locations included in the pilot study. Primary study extents are also indicated, which reveals convergence between reported site locations and survey coverage. Basemap Source: Google Earth 2018.

**Figure 4.**
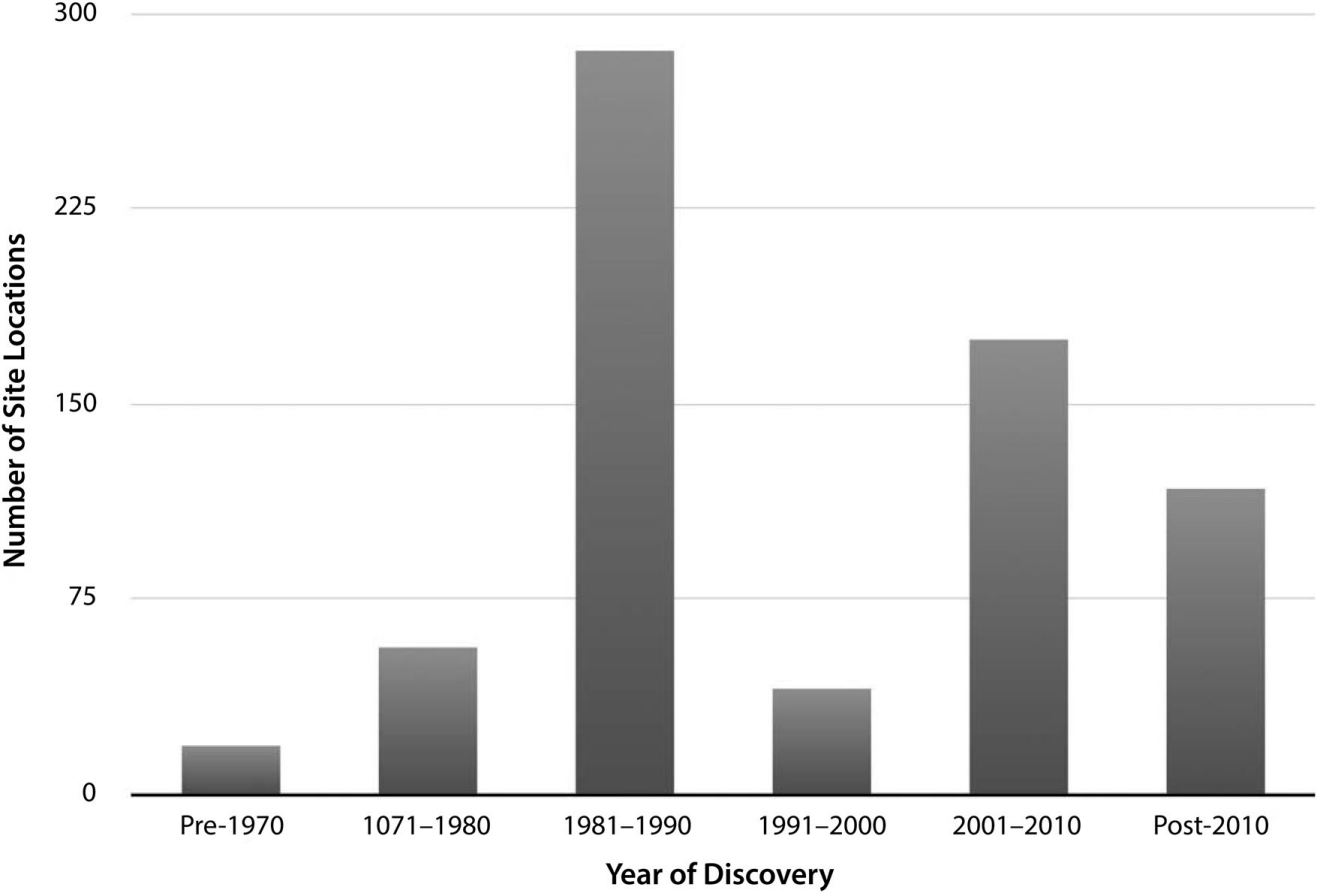
Bar graph depicting the number of sites reported in the decades following 1970.

**Figure 5.**
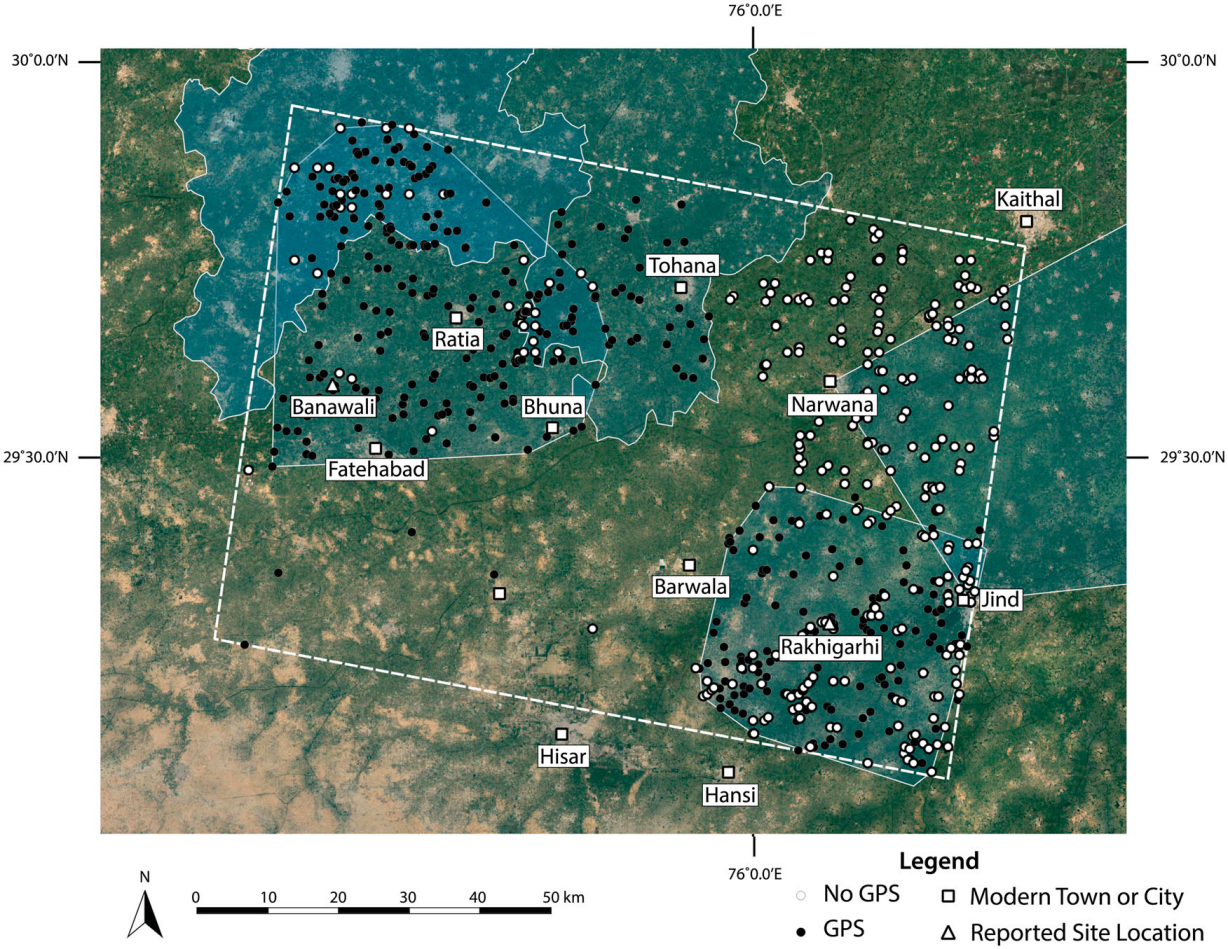
Distribution of site locations collected with or without the use of GPS. Primary study extents are also included, indicating that GPS has only been employed in recently surveyed areas. Basemap Source: Google Earth 2018.

The aggregate site location data assembled in the pilot database facilitated the development and testing of interpretations about Indus settlement density in northwestern India (figure 3). Most site locations were reported between 1981–1990, and there was a resurgence in archaeological survey that appears to have dramatically increased the number of reported site locations in the sample region following the year 2000 (figure 4). Unstandardized reporting conventions raise the need to examine the relationship between contemporary villages and archaeological sites in detail, as many coordinates in the database, especially in earlier reports, are known to reflect the location of nearby villages rather than the location of specific settlement mounds. The sample area included 695 previously reported site locations, 80% of which were reported with geographical coordinates that include degrees, minutes, and seconds (n = 554). However, there are also site locations that include seconds but are likely to be imprecise, with reported values of 00, 15, 30, or 45. Reassessment of these locations will be carried out in future stages of data consolidation and a sample of these locations will be updated after future fieldwork. Those reported without full geographical coordinates were typically documented in 2002 or earlier (n = 64), prior to the regular use of GPS. A negligible number (n = 14) of site locations appear to have been reported erroneously, either in recording of the site location in the field or in later re-publishing. Erroneous site locations have coordinates that appear to be incomplete or refer to locations that did not likely correspond to archaeological sites (as indicated in ESRI’s World Imagery Basemap). Though the great majority of site locations were reported with precise geographical coordinates, only 386 were likely collected with the aid of GPS (figure 5). It is clear that many of the reports in the northeastern quadrant of the study area were recorded without the assistance of GPS and may warrant re-investigation.

As survey coverage is not uniform, many sites likely remain to be discovered in areas that were ostensibly covered by secondary studies, but which may not actually have been surveyed extensively (figure 3). Around half (n = 372) of the site locations in the pilot database have only been reported once. Of those, 43% (n = 161) are site locations that pre-date the LWS surveys and do not appear to have been revisited or reconfirmed, while the remaining site locations (57%, n = 211) consist of new reports by the LWS or later surveys. This pattern of reporting has important implications for the identification of site concentrations: areas that have particularly high site densities may correspond to what Joshi and colleagues (1984) described as the Mature Harappan period’s economic pockets. Similar concentrations may remain unreported in areas that have not been recently surveyed, which is a possibility that warrants further testing.

**Figure 6.**
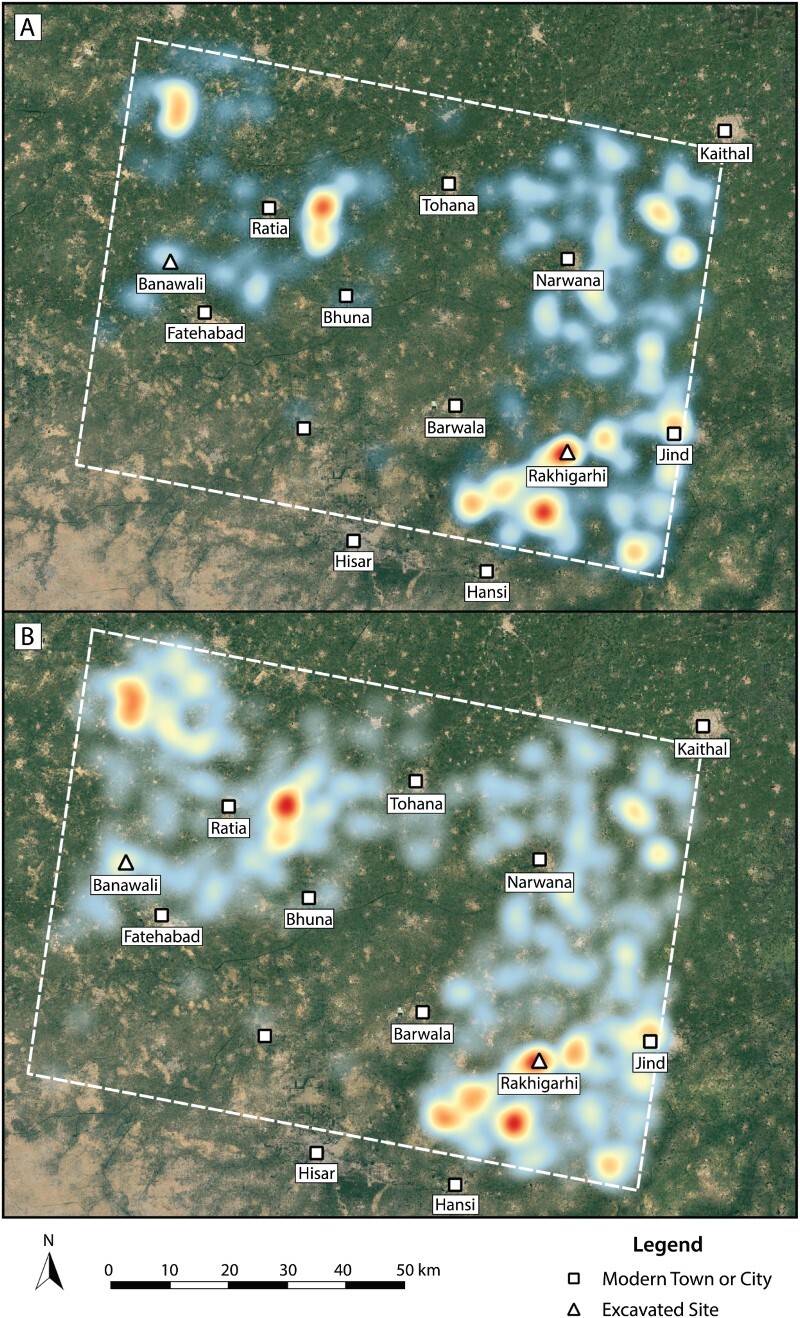
Density of site locations A) prior to and B) subsequent to 2009. Concentrations are depicted using a heat map color gradient between areas of high density (red) and low density (blue). Basemap Source: Google Earth 2018.

Recent efforts to improve survey coverage in northwestern India have transformed projections of site density in the study area, reinforcing previously identified patterns and revealing new ones. Figure 6 presents contrasting heat maps of location density for sites identified before and after 2009 for all periods. These were created using the Heatmap Plugin v0.2 for QGIS v2.18.2. The plugin was used to rasterize vector data derived from the pilot site location table (sorted by earliest year reported) using a radius value of 5 mm and a maximum automatic value. The best rendering quality setting was used, and the resulting raster layers were exported through a print composer that presented both side by side. These raster images assign each pixel a value according to the number of nearby site locations. The results of surveys prior to 2009 reveal several site location concentrations apparent in the dataset (figure 6b), including concentrations to the northwest and southeast of the modern city of Ratia in the northwestern quadrant of the study area and a slight concentration around the site of Banawali southwest of Ratia. A clear concentration was found around the site of Rakhigarhi, which appears to be aligned with linear concentrations of settlements extending toward the southwest. In line with this concentration near Rakigarhi are concentrations near Jind and northeast of the modern town of Hansi. In the northeastern quadrant, a further concentration appears northeast of the town of Narwana, not unlike those found in association with Rakhigarhi. Three concentrations in the northeastern quadrant are largely based on the findings of older surveys (Suraj Bhan 1975; Suraj Bhan and Shaffer 1978). Recent surveys have enhanced the clarity of these findings (figure 6b). Given that increased survey efforts confirmed previously identified patterns, it will be critical for future surveys to reassess the concentrations identified in the northeastern quadrant, which have not yet been revisited.

**Figure 7.**
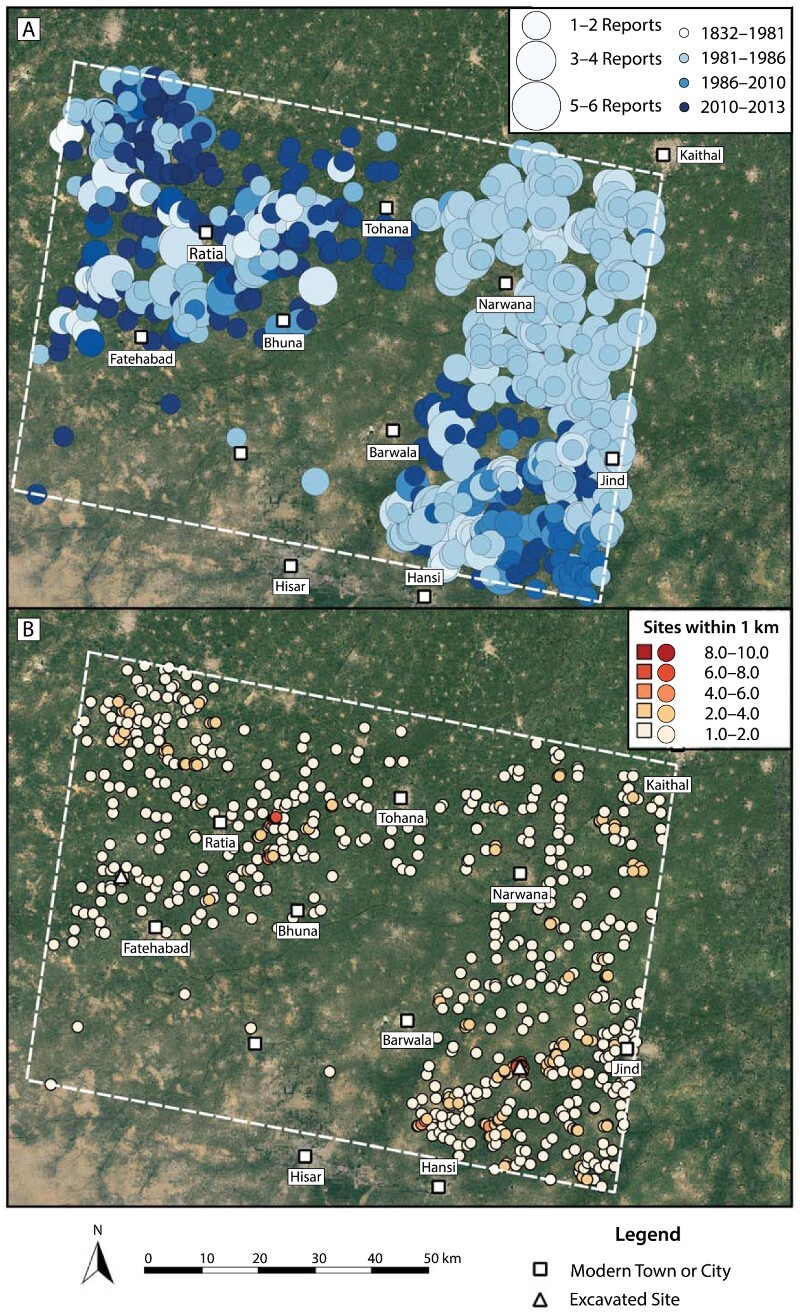
Analysis of site location characteristics. Site locations depicted according to: A) number of times reported and year of earliest report; and B) proximity to other sites, defined by the number of sites within one kilometer. Basemap Source: Google Earth 2018.

It is unclear whether areas with few reported site locations, such as between the LWS survey areas, were in fact thinly occupied, or whether they simply require additional study. There is a gap in survey coverage within the southwestern quadrant of the sample area, extending around today’s city of Hisar and the village of Barwala. Site density in the northeastern corner of the study area, however, is similar to that seen in the areas covered by the LWS surveys. While reported sites in the northeastern quadrant of the survey area are numerous, none of the locations were collected with the assistance of GPS (figure 5). The site locations reported in the northeastern quadrant of the sample area are nonetheless characterized by a clear pattern. Figure 7A depicts each site according to the number of times it has been reported (as increasing size) and the earliest year of its report (darker blue is more recent). Those in the northeastern quadrant have been re-reported often, and although their original reports are quite early (Suraj Bhan and Shaffer 1978), they have not been revisited. While some concentrations of sites in the northwestern and southeastern quadrants have a similar pattern in reporting, they have been surveyed more intensively in recent years.

The northeastern quadrant exhibits patterns in site proximity that are similar to those in the LWS survey areas (figure 7b). Assuming a settlement’s overall spatial plan was approximately circular, a buffer of 1 km around a site location would encapsulate the entire area of even the largest Indus cities (Mohenjo-daro’s largest reported area exceeds 200 hectares [Jansen 1993]). Calculating the number of site locations that fall within 1 km of one another reveals that each site is proximal to a mean of two others. Twenty-eight site locations are within 1 km of 5 other site locations, and four are within a kilometer of more than six other sites. In the more intensively surveyed northwestern and southeastern quadrants, high-proximity sites are often associated with major settlements, such as Rakhigarhi and Banawali. The northeastern quadrant, in contrast, has not benefited from recent survey efforts, and yet high proximity site locations exist within this quadrant.

**Figure 8.**
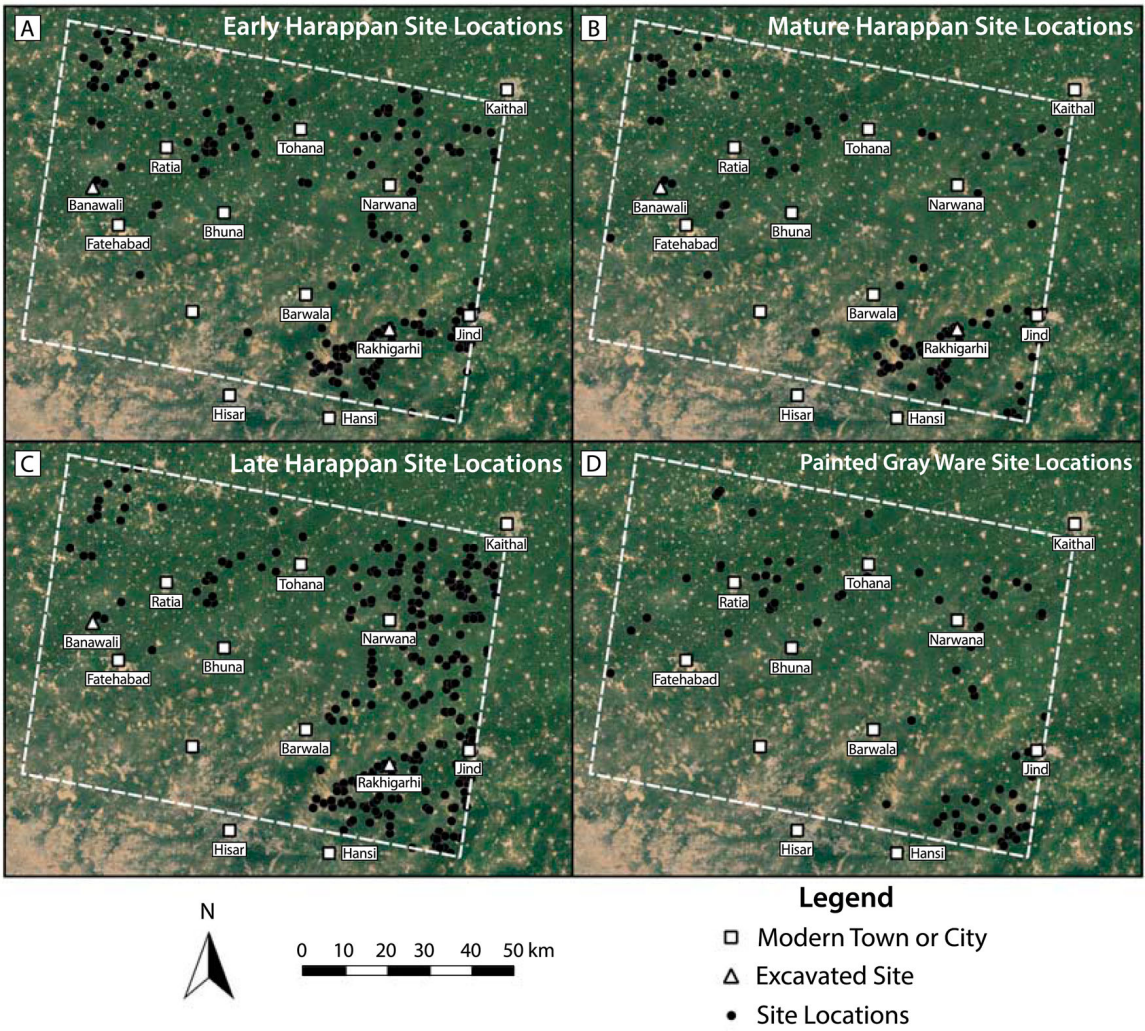
Changes in site location distribution through time. Basemap Source: Google Earth 2018.

**Figure 9.**
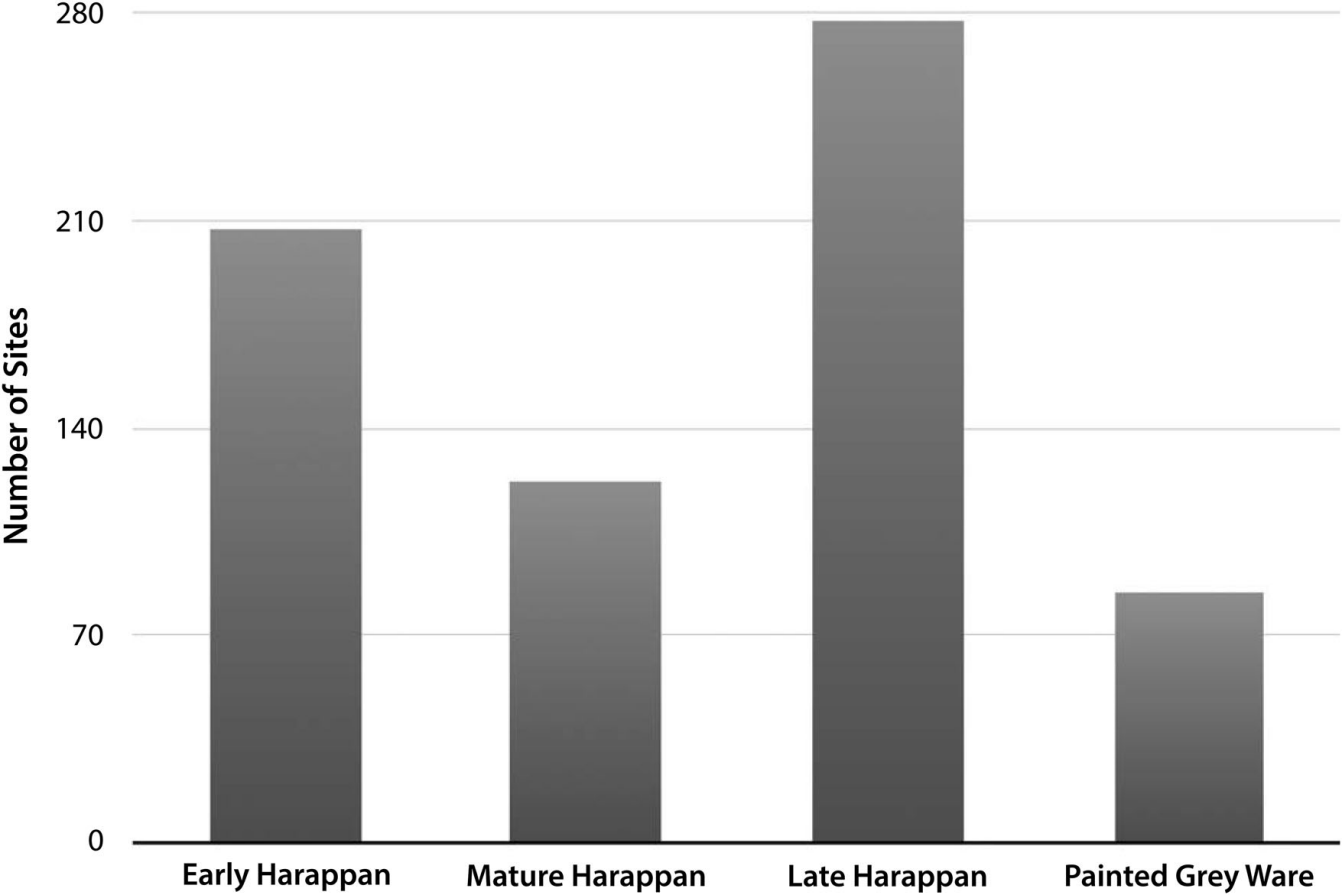
Bar graph derived from the number of reported sites belonging to particular chronological periods.

Reported chronological data reveals diachronic changes in the locations that were favored for settlement as people left Indus cities beyond (figure 8). Just over half of the site locations in the sample (n = 343) have been characterized as Early (n = 207), Mature (n = 122), and/or Late Harappan (n = 278) (figure 9). Many site locations have components that post-date the Indus Civilization, with materials that belong to the Painted Gray Ware (n = 84), Early Historic (n = 245), and/or Medieval (n = 221) periods. These figures support the hypothesis that the overall number of settlements decreased during the Mature Harappan period and increased as the major cities were depopulated after ca*.* 1900 b.c. (figure 9). The spatial dimensions of these trends support previous research on settlement density and northwestern India, and can be used to develop new research questions.

## Discussion

This paper supports the interpretation that the number of settlements in northwestern India decreased during the Indus Civilization’s Mature Harappan period. Notably, the LWS surveys did not document increases in post-urban occupation in either of the areas of the primary surveys, which suggests that any increases occurred elsewhere (Petrie et al. 2017). Settlement increases may have occurred in the northeastern quadrant of the sample area, contributing to the increasing of the settlement density of northwestern India in the Late Harappan and Painted Gray Ware periods.

It is reasonable to state that sites that have been characterized as Early Harappan were evenly distributed within surveyed regions, which is the view proposed by Chakrabarti and Saini (2009) and supported by subsequent projects (Dangi 2011). Gaps in the distribution of Early Harappan sites around the future urban center of Rakhigarhi, and concentrations in the distribution of GHS sites in the northwestern corner of the sample area have, however, been detected (Singh et al. 2010: 41, 2011: 100). Early Harappan settlements thus appear to have been numerous, but tended to be some distance apart from one another. This apparent pattern may be the result of data quality, as the most widely distributed site locations appear to correspond to older surveys (figure 7a), but the patterns are not mutually exclusive, and their co-occurrence suggests that the people who established these early settlements did not adopt a single approach to obtaining or accessing water. Petrie and colleagues (2017) have suggested that this distribution likely set the stage for the Indus Civilization’s later emergence, positioning settlements to take advantage of a wide variety of water sources.

The Mature Harappan period saw an overall reduction in the absolute number of site locations (figure 9). There is no consensus as to whether the emergence of Indus cities required dramatic changes in water use. Chakrabarti (1988, 1999: 327) has long argued that canal based irrigation may have been important, and there is evidence for major water storage facilities at sites like Dholavira (Bisht 2005; Wright 2010). Others have proposed that Indus settlements had a wide variety of low-cost irrigation techniques at their disposal (Miller 2006, 2015; Wright 2010: 33–34; Petrie 2017), but our understanding of water supply in Indus period northwestern India remains nascent. That there are fewer site locations in the Mature Harappan period than in the Early Harappan period indicates a general concentration of settlement in specific areas (figure 8b). The pattern appears to have been variable, however, and the reduction of settlement in the northwestern corner of the sample area (Singh et al. 2011: 101) was more pronounced than the reduction in the number of Mature Harappan sites near Rakhigarhi (Singh et al. 2010: 46; Petrie et al. 2017). Given the apparent diversity in cropping practices that is evident in northwestern India’s Mature Harappan period (Petrie 2017; Petrie et al. 2016, 2017; Bates et al. 2017a, 2017b; Petrie and Bates 2017), and the problematic linkage between site location and watercourses that has often been assumed (see review by Petrie and colleagues [2017] and Singh and colleagues [2010: 44, 2011: 102]), it is essential to further investigate the socio-economic and environmental dynamics that contributed to this concentration of settlement during the height of the Indus Civilization.

The Late Harappan period marked a return to the widespread distribution of site locations observed during the Early Harappan period (figure 8a, c). This reassessment has confirmed that around Rakhigarhi, Late Harappan settlement site locations are more numerous than, but generally proximal to, their Mature Harappan predecessors, which is a pattern previously identified by Singh and colleagues (2010: 42). The results presented here, however, confirm that site locations in the northwestern corner of the sample area are dramatically reduced overall in the Late Harappan period (Singh et al. 2011; Petrie et al. 2017). The northeastern quadrant of the sample area appears to have been densely occupied in the Early Harappan period and re-occupied later. There thus appears to have been a shift in settlement locus from the northwestern to the northeast of the sample area during the closing years of the Mature Harappan period (figure 8b), and potentially also movement of populations into the northeast from outside of the study area. It has been argued that this particular area of the plain may have had more reliable monsoon rainfall (Petrie 2017; Petrie et al. 2017). A shift toward this part of the plain may have been a key strategy for building resilience in the changing climatic conditions that characterize the end of the Mature Harappan period (Petrie et al. 2017). However, it remains unclear to what extent this Late Harappan shift towards the northeastern quadrant of the study area may be an artifact of early methods and assumptions.

Determining the veracity of the Late Harappan shift is critical, considering that in the subsequent periods (figure 8c–d) no site locations have yet been reported in the northeastern quadrant of the sample area. This, again, may reflect survey methods, the chronological breadth of surveys, and/or the research interests of surveyors, rather than an actual absence of sites. There are, however, numerous reports of Painted Gray Ware sites in the northwestern quadrant, and a further increase in settlement there in the Early Historic period (Singh et al. 2011). It is notable that many of these later sites contribute to the growing concentration of sites stretching from immediately east of Ratia to just north of Fatehabad, which is shown to striking effect in Figure 6a. The distribution of Painted Gray Ware sites also breaks with the concentration of Late Harappan sites near Rakhigarhi (Singh et al. 2010: 46).

Prior to 2009, a total of 455 sites had been reported within the sample area. This number has increased substantially since then, increasing the total reported site locations while increasing survey coverage in less than half of the sampled area. If similar quantities of new site locations are reported throughout the entire sample extent, the number of total site locations could well increase another twofold. Future data integration work will address these issues, as will iterative phases of fieldwork to ground truth and update site location data. Moreover, the category of “site” needs to be expanded to specify different kinds of archaeological phenomena in northwestern India, and it is essential to conduct complementary intensive surveys at individual sites, systematically assessing surface materials to identify and delineate the specific spatial distribution of different classes of artifacts and features, an approach which has yielded considerable insights into social relations between the Indus city of Harappa and its surrounding settlements in Pakistan’s Punjab (Wright et al. 2001, 2003). Adopting these techniques could contribute new regional perspectives on patterns in material culture that are unbound by the site concept (Kantner 2008; Howey and Burg 2017).

The ptr_id table has provided a means of tentatively assessing certainty in site location datasets from northwestern India. At this stage, the pilot database speaks primarily to the archaeological significance and geographical precision of site location reports, though continued database development will allow the assessment of variables such as site boundary certainty and, thus, site size. There remain many unpublished and, at present, inaccessible site location datasets that must be digitized and added to the database. As this database grows and the findings presented here are confirmed (or refuted) through further fieldwork, it will be possible to identify further gradations of certainty in site location data, and test hypotheses at larger scales.

The study reveals the necessity of examining the silos in which archaeological survey data are generated and analyzed. Projecting site locations merely as dots on a map can lure researchers into thinking they understand previous settlement patterns better than they do, while site locations that remain more or less unmoved after multiple on the ground surveys are of particular value. The Indus Civilization in northwestern India is particularly different in this regard, as it takes many different survey datasets to understand the Indus Civilization’s settlement distribution, incorporating some areas that have been surveyed again and again. This very fact means that certain trends in settlement are surer than others. Further investigation of the Indus Civilization’s signature landscapes also has the potential to enhance alternative models of social complexity, revealing how heterarchical social relations may have materialized and supported social relations across vast and varied environments.

## Conclusions

Archaeological survey data are essential for understanding the dynamics of social complexity. Identifying the signature landscapes that materialized the prevailing social processes that underpinned these dynamics requires large scale analysis that exceed the boundaries of most individual field survey projects. By integrating site location data from multiple projects, this paper offers new support for the interpretation that northwestern India comprised one or more of the Indus Civilization’s signature landscapes, where settlement densities chart trajectories of urbanization and de-urbanization, involving agglomeration and dispersal into areas with suitably favorable environmental conditions. Site location concentrations appear to generally correspond to previous survey coverage, and there has been an overall underestimation of northwestern India’s settlement density across both time and space. There remain many areas where systematic surveys are needed, such as the broad area between the LWS surveys, and many areas would benefit from re-visitation and re-evaluation, such as the site locations reported in the northeastern quadrant of the study area. An extensively occupied landscape appears to have emerged during the Early Harappan period and was largely re-occupied during the Late Harappan period, as there appears to have been a displacement of settlement into specific parts of the plain. It remains necessary to test the veracity of this re-occupation by reassessing sites located in the northeastern corner of the surveyed area and closing gaps in survey coverage. Engaging in such reassessment will contribute to research on the signature landscapes that inform scholarly understanding of urbanization and de-urbanization and the impact of variable and changing environments on settlement distributions in the past.

## Supplementary Material

Green_Supplemental_Material_RFP.csv
